# Mediator MED23 controls oligodendrogenesis and myelination by modulating Sp1/P300-directed gene programs

**DOI:** 10.1038/s41421-024-00730-8

**Published:** 2024-10-15

**Authors:** Shuai Zhang, Xue Feng, Chong-Hui Li, Yuan-Ming Zheng, Meng-Ya Wang, Jun-Jie Li, Yun-Peng Dai, Naihe Jing, Jia-Wei Zhou, Gang Wang

**Affiliations:** 1 State Key Laboratory of Genetic Engineering, School of Life Sciences and Zhongshan Hospital, Fudan University, Shanghai, China; 2 Laboratory Animal Resource Center, Fudan University, Shanghai, China; 3 State Key Laboratory of Cell Biology, Center for Excellence in Molecular Cell Science, Shanghai Institute of Biochemistry and Cell Biology, Chinese Academy of Sciences, Shanghai, China; 4 School of Life Science and Technology, ShanghaiTech University, Shanghai, China; 5 Guangzhou Laboratory, Guangzhou, Guangdong China; 6 Institute of Neuroscience, State Key Laboratory of Neuroscience, CAS Center for Excellence in Brain Science and Intelligence Technology, Chinese Academy of Sciences, Shanghai, China

**Keywords:** Mechanisms of disease, Transcription, Developmental biology, Neural stem cells

## Abstract

Gaining the molecular understanding for myelination development and regeneration has been a long-standing goal in neurological research. Mutations in the transcription cofactor Mediator Med23 subunit are often associated with intellectual disability and white matter defects, although the precise functions and mechanisms of Mediator in myelination remain unclear. In this study, we generated a mouse model carrying an Med23^Q649R^ mutation that has been identified in a patient with hypomyelination features. The MED23^Q649R^ mouse model develops white matter thinning and cognitive decline, mimicking common clinical phenotypes. Further, oligodendrocyte-lineage specific *Med23* knockout mice verified the important function of MED23 in regulating central nervous system myelination and postinjury remyelination. Utilizing the in vitro cellular differentiation assay, we found that the oligodendrocyte progenitor cells, either carrying the Q649R mutation or lacking Med23, exhibit significant deficits in their capacity to differentiate into mature oligodendrocytes. Gene profiling combined with reporter assays demonstrated that Mediator Med23 controls Sp1-directed gene programs related to oligodendrocyte differentiation and cholesterol metabolism. Integrative analysis demonstrated that Med23 modulates the P300 binding to Sp1-targeted genes, thus orchestrating the H3K27 acetylation and enhancer activation for the oligodendrocyte lineage progression. Collectively, our findings identified the critical role for the Mediator Med23 in oligodendrocyte fate determination and provide mechanistic insights into the myelination pathogenesis associated with MED23 mutations.

## Introduction

Disruption of myelin, the multilayered sheath enveloping axons, in the central nervous system (CNS) leads to various neurological diseases, such as multiple sclerosis and leukodystrophies^1,2^. Oligodendrocytes (OLs), the myelin-forming cells in the CNS, arise from self-renewing oligodendrocyte precursor cells (OPCs), which populate the entire CNS and subsequently extend numerous processes to myelinate axons^3,4^. OL lineage progression is accompanied by profound changes in both morphology transition and gene expression that require tight coordination mediated by transcriptional regulation and epigenetic programming^5,6^. Therefore, elucidating key regulatory factors involved in OL development is critical to understanding the cellular and developmental biology of myelin production and regeneration.

Mediator complex serves as a control panel to converge developmental and environmental signaling onto the basal transcriptional machinery^7,8^. Variants in Mediator subunit genes have been implicated in the pathogenesis of various human diseases, manifesting as developmental defects, neurological disorders, and tumorigenesis^9,10^. Moreover, recent genetic studies have shown that mutations in the human *MED23* gene lead to the acquisition of a spectrum of phenotypes, including intellectual disability and neurological dysfunction, most of which are accompanied by abnormal CNS myelin development, such as thinning of the corpus callosum and temporal lobe hypomyelination^11–13^. However, the mechanisms underlying these neurological disorders, particularly white matter defects suffered by children, are poorly defined.

Here, we developed a mouse model to understand the consequences of MED23^Q646R^ mutation in vivo, inspired by a patient with a novel homozygous pathogenic *MED23* variant. The mutant mice recapitulated the clinical phenotypes of hypomyelination and cognitive impairment. In vitro, OPCs carrying the Med23^Q649R^ mutation failed to initiate the formation of arborized cytoarchitecture and express maturation genes. To further explore the functions and mechanisms of Mediator Med23 in OL development, we generated the OL lineage-specific *Med23* knockout (KO) mice, which displayed widespread myelin deficits and aberrant myelin regeneration. Using integrative approaches, we demonstrated a mechanistic role for Med23 in modulating the cooperation between Sp1 and P300 in active maintenance of myelination-promoting gene promoters and enhancers. Overall, our findings provide insight into the mechanistic underpinnings of the MED23-associated neurological disorders and enlighten the possible therapeutic interference strategies.

## Results

### Med23^Q649R^ mutant mice display myelin defects together with abnormal cognitive behaviors

**Fig. 1:**
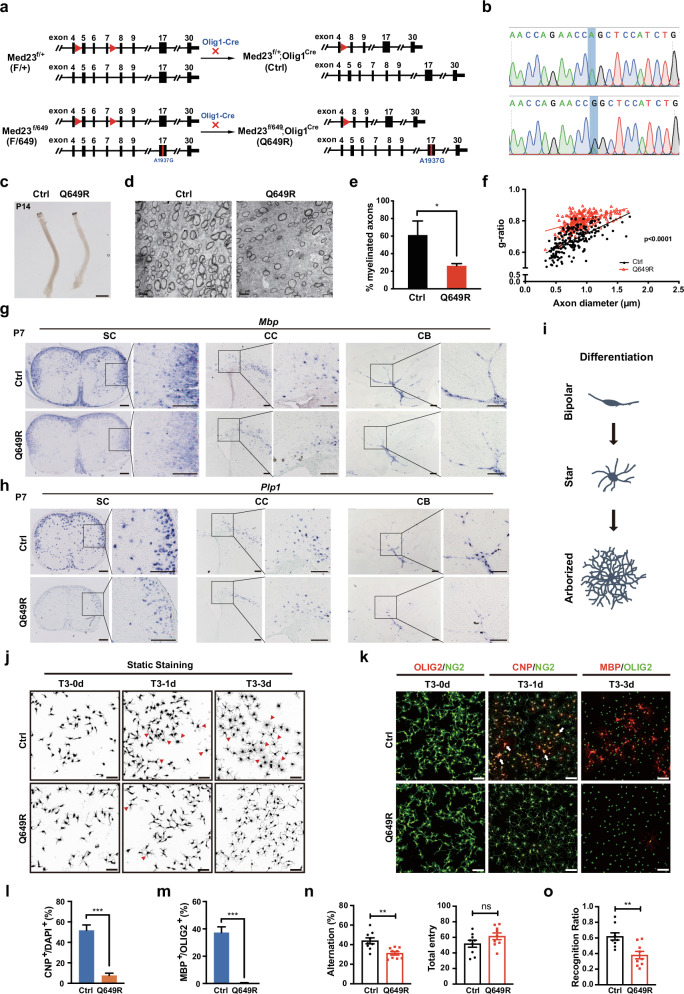
Med23^Q649R^ mutation disrupts OL maturation. **a** Strategy for the generation of OL lineage-specific Med23^Q649R^ mutant mice. **b** Sanger sequencing of the Q649R mutation on the *Med23* mutant allele. **c** Photographs showing optic nerves of control and Med23^Q649R^ mice at P14. Scale bar: 1 mm. **d**–**f** Representative electron micrographs of optic nerves in control and *Med23*cKO mutants at P14. Scale bars: 2 μm. The percentage of myelinated axons is shown in **e**, and the myelin g-ratio scatterplot vs axon diameters is quantified in **f** (*n* = 3). **g**, **h** In situ hybridization for *Mbp* and *Plp1* in the spinal cord (SC), forebrain (FB), and cerebellum (CB) from control and Med23^Q649R^ mice at P7. Scale bars: 100 μm. **i** Cartoon depicting stereotype stages of OL morphology changes during differentiation in culture. **j** Representative micrographs showing control and Med23^Q649R^ OLs that were in differentiation medium for 0 day and 3 days in culture. Scale bars: 50 μm. **k** OPCs isolated from control and Med23^Q649R^ mice were cultured in differentiation medium for 3 days. The cells were stained for Olig2 (red) and NG2 (green) at day 0, CNP (red) and NG2 (green) at day 1, and MBP (red) and Olig2 (green) at day 3. Scale bars: 50 μm. **l** Quantification of the percentage of CNP^+^ cells among total DAPI^+^ cells in control and Med23^Q649R^ OLs after 1 day of differentiation (*n* = 3). **m** Quantification of the percentage of MBP^+^ cells among total DAPI^+^ cells in control and Med23^Q649R^ OLs after 3 days of differentiation (*n* = 3). **n** Comparison of spontaneous alternation percentage (left) and total entry (right) in the Y-maze test between the control and Med23^Q649R^ adult mice (*n* = 9). **o** Comparison of the preference index for a novel object in the 2-object novel object recognition test between the control and Med23^Q649R^ adult mice (*n* = 9). The data are presented as the mean ± SEM; two-tailed unpaired Student’s *t*-test; ns > 0.05; *P < 0.05, **P < 0.01, and ***P < 0.001.

Recent genomic data linked the human *MED23* mutations to dysmyelination features^12^, implying that *MED23* variants may confer high susceptibility to myelination abnormalities. Notably, one patient with a point mutation in the *MED23* gene (GRCh38.p12; chr6:131603042T>C; c. A1937G; p. Q646R) was reported to manifest hypomyelination features^12^. To investigate the pathogenic effects and mechanisms of the human MED23^Q646R^ mutation, we determined to generate mutant mice harboring the Med23^Q649R^ (Q646R in humans and Q649R in mice) mutation utilizing the CRISPR-Cas9 technology. However, repeated efforts failed to generate homozygous mutant mouse lines, as the homozygous mutants (Med23 ^Q649R/Q649R^) were embryonic lethal (E9.5) (Supplementary Fig. S1a). The mRNA expression of *Med23* in OL-lineage was not affected by Med23^Q649R^ mutation (Supplementary Fig. S1c). Moreover, the expression levels of Med23, detected by western blot, were comparable between wild-type (WT) and homozygous mutant embryonic stem cells (ESCs) (Supplementary Fig. S1d), suggesting that the Med23^Q649R^ mutation may not disrupt Med23 expression. To acquire the survival mutant mice for further analysis, we have made great efforts to generate the OL lineage-specific *Med23* mutant mice carrying the Med23^Q649R^ missense mutation. First, the *Med23*^*fl/fl*^ line^14^ was intercrossed with viable *Med23*^*+/Q649R*^ mutants to generate compound heterozygotes (*Med23*^*fl/Q649R*^), of which the optic nerves seemed to be normally myelinated (Supplementary Fig. S1b). Then the *Olig1-cre*^15^ was introduced to generate the lineage-specific point mutant mouse model (*Med23*^*fl/Q649R*^*;Olig1-Cre*, i.e., Med23^Q649R^), which carries a single Q649R allele of *Med23* in OL lineage (depicted in Fig. 1a). Med23^Q649R^ animals were born at the expected Mendelian ratio and were of normal body size. Genotyping revealed a point mutation in one of the *Med23* alleles, which caused a single amino acid change; that is, glutamine was replaced with arginine, in exon 17 of Med23 (Fig. 1b). We then compared Med23^Q649R^ mice with their littermates (*Med23*^*fl/+*^*;Olig1-Cre*, i.e., Control) to examine the effects of the Med23^Q649R^ mutation. Notably, the optic nerves in the Med23^Q649R^ mice appeared to be translucent compared to the control (Fig. 1c), and the electron microscopy (EM) analysis revealed that the percentage of myelinated axons and the thicknesses of myelin sheaths, two indexes to measure myelin assembly, were markedly reduced in the optic nerves (Fig. 1d–f). Moreover, in situ hybridization showed that the expression of myelin genes such as *Mbp* and *Plp1* was dramatically decreased in the forebrain, spinal cord, and cerebellum of the Med23^Q649R^ mice at postnatal day 7 (P7, Fig. 1g, h). However, Med23^Q649R^ mutant allele did not lead to notable development alterations in astrocyte, neuron, and microglia (Supplementary Fig. S1e). Together, these results suggested that CNS myelinogenesis was disrupted by Med23^Q649R^ mutation, and the Med23^Q649R^ mice recapitulated the myelination abnormalities observed in brains of Med23 mutation carriers.

We then used the in vitro differentiation system to investigate the effects of this mutation on OL development. OPCs isolated from the Med23^Q649R^ mice or control mice were induced to differentiate by triiodothyronine (T3) for 3 days. This differentiation process progressed through three consecutive stages: OPCs (0 day), immature OLs (iOLs at 1 day), and mature OLs (mOLs at 3 days). Morphology and gene expression changes of the differentiating cells were analyzed at the representative time points. Both Med23^Q649R^ and control OPCs displayed similar bipolar morphology (Fig. 1j) when plated in a growth medium containing PDGF and NT3, suggesting that Med23^Q649R^ mutation did not change the OPC identity. Then the cells were subjected to T3-induced differentiation. Previous studies have demonstrated that OPC differentiation is accompanied by morphological conversion from bipolar precursor to mOLs with highly branched processes^16^ (depicted in Fig. 1i). Here, static staining showed that most of the control OPCs differentiated in a stereotypic manner with highly ramified processes after T3 treatment for 3 days, a clear manifestation of maturation. In comparison, the Med23^Q649R^ cells failed to form an arborized cytoarchitecture, and they produced shorter branches with fewer processes, indicating that Med23^Q649R^ mutation blocked OPC process extension and prevented morphological maturation (Fig. 1j). Morphological transformation during OL development is always accompanied with the cell surface marker changes^4^. Immunostaining for early (CNP^+^) and late (MBP^+^) OL maturation markers throughout the differentiation process demonstrated deficient expression of mature OL markers in Med23^Q649R^ cells compared with that in the control cells (Fig. 1k–m). These observations demonstrated that the Med23^Q649R^ mutation was sufficient to interrupt OL differentiation and myelin formation.

Recent studies implicated myelin abnormalities in cognitive impairment^17–19^. We then evaluated whether the Med23^Q649R^-associated myelin changes influence cognitive function through two classical behavioral tests. The Y-maze test was used to evaluate spatial, retroactive working memory abilities^20,21^. The Med23^Q649R^ mice showed decreased spontaneous alternations in the Y-maze test (Fig. 1n), indicating their worse working memory to recall which arms already visited. We confirmed the learning and memory deficits of Med23^Q649R^ mice by performing the novel object recognition test (NORT) to assess attention and short-term memory^19,22^. The control mice spent more time exploring the novel object than the familiar object, but by contrast, the Med23^Q649R^ mice failed to discriminate between the two objects and therefore spent less time with the novel object (Fig. 1o). Overall, these data support that the Med23^R649Q^ mutation compromised oligodendrogenesis and CNS myelination, which may have contributed to the pathology of the observed cognitive impairments.

### OL lineage-specific Med23 ablation results in myelination defects

**Fig. 2:**
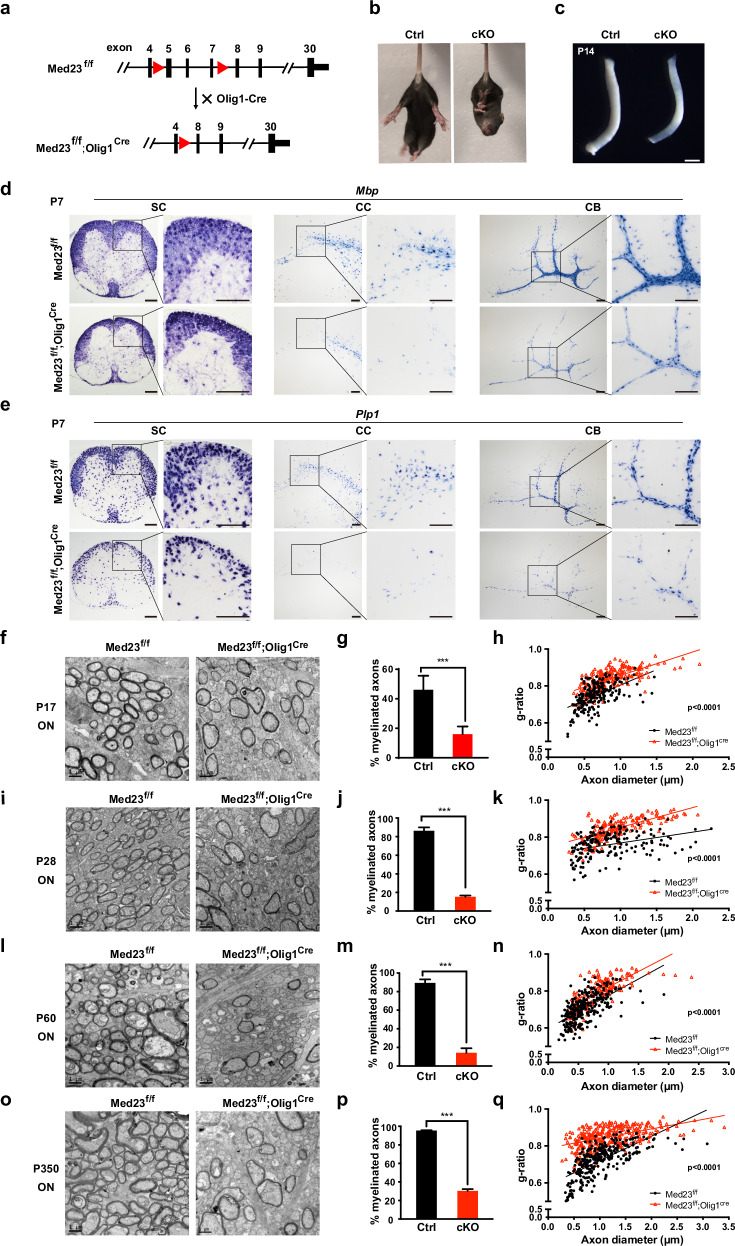
Mice lacking Med23 in the OL lineage display CNS hypomyelination. **a** Strategy for the generation of the OL lineage-specific Med23 deletion in mice. **b** Tail suspension test for Ctrl (*Med23*^*fl/fl*^) and *Med23*cKO (*Med23*^*fl/fl*^*;Olig1-Cre*^*+/–*^) mice at P14. **c** Appearance of optic nerves in Ctrl and *Med23*cKO littermates at P14. Scale bar: 1 mm. **d**, **e** In situ hybridization for *Mbp* and *Plp1* in the spinal cord (SC), corpus callosum (CC), and cerebellum (CB) from Ctrl and *Med23*cKO mice at P7. Scale bars: 100 μm. **f**, **i**, **l**, **o** Representative electron micrographs of optic nerves in Ctrl and *Med23*cKO mice at P17, P28, P60, and P350. Scale bars: 1 μm. **g**, **j**, **m**, **p** The percentage of myelinated axons in Ctrl and *Med23*cKO optic nerves at the indicated stages. The data are presented as the mean ± SEM; *n* = 3 animals/genotype. Two-tailed unpaired Student’s *t*-test, ***P < 0.001. **h**, **k**, **n**, **q** Myelin g-ratio scatterplot vs axon diameters of the optic nerves in Ctrl and *Med23*cKO at indicated stages. *n* = 3 animals/genotype. (≥ 70 myelinating axons were counted for each mouse). Two-tailed unpaired Student’s *t*-test, *P* < 0.0001.

To gain better understanding of MED23 on the myelination, we explore the opportunity of constructing a complete ablation of Med23 in the OL lineage, using the *Med23*^*fl/fl*^ mice crossed to OL lineage-specific *Olig1-Cre* line^15^ (Fig. 2a). The efficiency of Med23 deletion was confirmed by western blot analysis using primary OPC lysates from conditional *Med23*-KO mice (*Med23*^*fl/fl*^*;Olig1-Cre*^*+/–*^, i.e., *Med23*cKO) and their control littermates (*Med23*^*fl/fl*^) (Supplementary Fig. S2a). *Med23*cKO mice were born following expected Mendelian ratio and were not overtly distinguishable from their control littermates. However, from postnatal week 2, the *Med23*cKO mice exhibited an abnormal limb clasping phenotype, a sign of neurological disorders (Fig. 2b). Notably, the optic nerves in *Med23*cKO mice appeared to be translucent compared to the control (Fig. 2c), indicating myelin deficit. The FluoroMyelin stain analysis showed that the myelination in the corpus callosum, cerebellum, and spinal cord was significantly reduced in *Med23*cKO mice at postnatal day 14 (P14) (Supplementary Fig. S2b). Furthermore, the reduced myelin gene expression in the *Med23*cKO mice was revealed by in situ hybridization, as manifested by substantially lower *Mbp* and *Plp1* expression in the aforementioned CNS regions at P7 (Fig. 2d, e) and P14 (Supplementary Fig. S2c).

In light of these findings, we used EM to further examine the ultrastructure of myelin sheaths in the optic nerves and spinal cord and found dramatically compromised axonal ensheathment in the CNS of *Med23*cKO animals at P17 (Fig. 2f–h; Supplementary Fig. S2d–f). Consistently, the optic nerves of the *Med23*cKO mice displayed myelin abnormalities, with increased g-ratios and nonmyelinated axon percentages at P28, P60, and P350 (Fig. 2i–q). These data indicated that Med23 deletion in the OL lineage resulted in CNS myelination deficits, which persist into adulthood. Together, these results, consistent with the acquisition of the Med23^Q649R^ phenotype, demonstrate that Med23 is essential for CNS myelination.

### Med23 is required for remyelination after demyelination injury

**Fig. 3:**
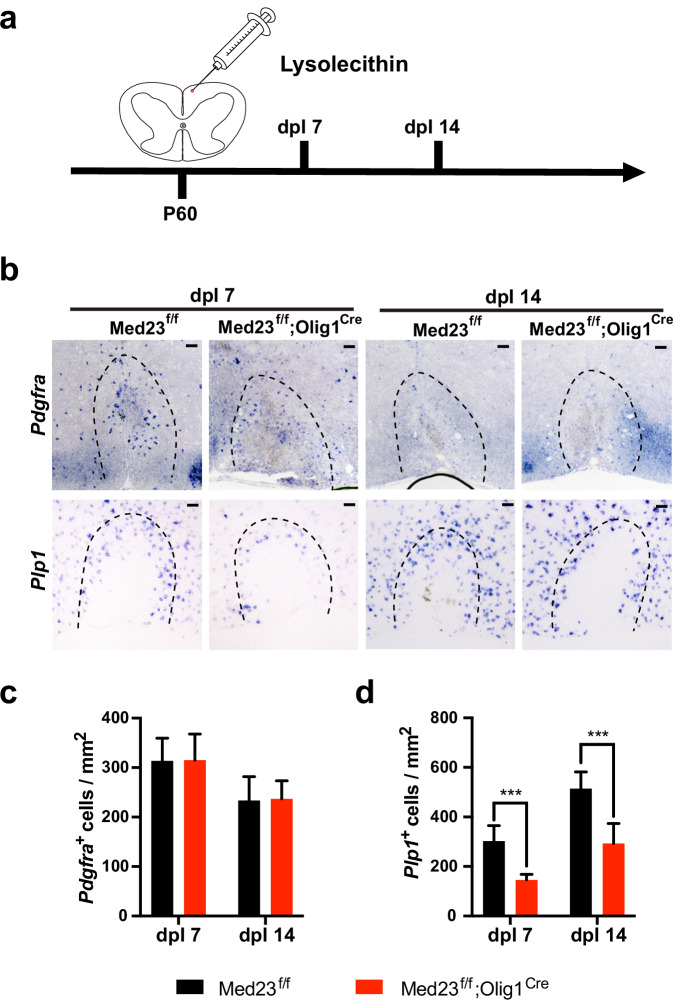
Med23 ablation impairs remyelination capacity in the LPC injury mouse model. **a** Diagram showing the LPC injection schedule. **b** In situ hybridization for *Pdgfra* and *Plp1* in spinal cord lesions from P60 Ctrl and *Med23*cKO mice at dpl 7 and dpl 14. Scale bars: 50 μm. **c**, **d** Quantification of the number of *Pdgfra*^+^ OPCs (**c**) and mature *Plp1*^+^ OLs (**d**) in spinal cord lesions from P60 Ctrl and *Med23*cKO mice at 7 and 14 dpl. The data are presented as the mean ± SEM; *n* = 3 animals/genotype. Two-tailed unpaired Student’s *t*-test, ***P < 0.001.

Remyelination is the regenerative process during which new myelin sheaths are restored to axons following pathological demyelination^23^. Given the essential role of Med23 in myelin development, we hypothesized that Med23 might be involved in the remyelination process following lysolecithin (LPC)-induced demyelination. In this model, a spontaneous myelin regeneration process is initiated with an OPC recruitment phase that continues for ~7 days post-LPC (dpl 7) lesion induction, followed by a remyelinating phase around dpl 14^24^. Then we analyzed the regenerated OPCs and OLs in the lesions at the two-time points (Fig. 3a). At dpl 7 and 14, the number of emerging PDGFRα^+^ OPCs in spinal cords with LPC-induced lesions was comparable between the *Med23*cKO and control mice (Fig. 3b, c); however, the number of *Plp1*^+^ mature OLs was markedly reduced in the lesions of *Med23*cKO compared with control mice (Fig. 3b, d). These findings suggest that Med23 deficiency does not change OPC formation but causes a reduction in OL regeneration in the lesion, thus compromising remyelination.

### Med23 deficiency leads to a reduction in the number of mature OLs in vivo

**Fig. 4:**
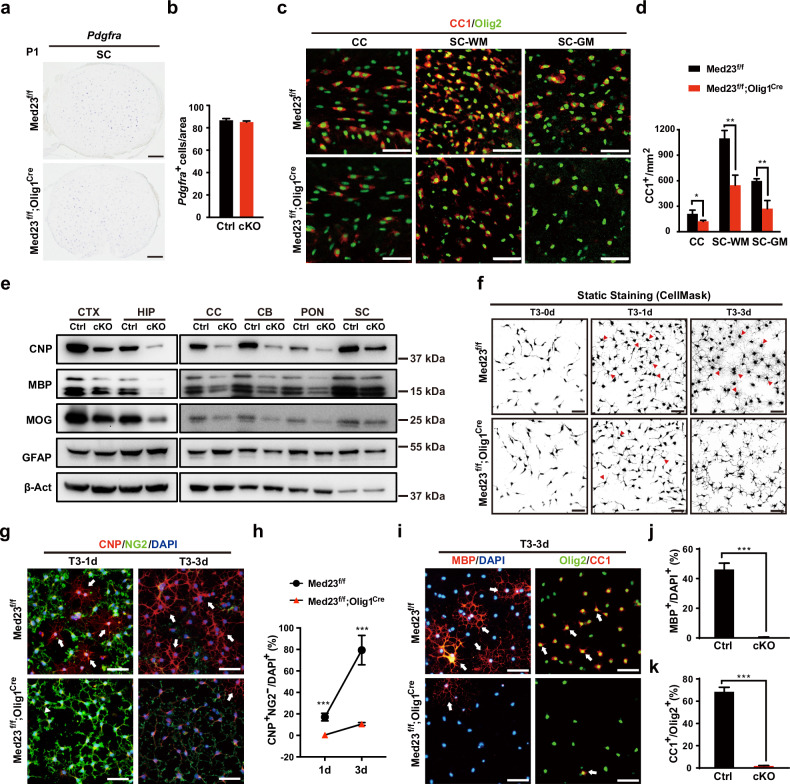
Med23 deletion impairs OL maturation. **a** In situ hybridization for *Pdgfra* in spinal cord sections of Ctrl and *Med23*cKO mice at P1. Scale bars: 100 μm. **b** Quantification of *Pdgfra*^+^ cells per area of the spinal cord in Ctrl and *Med23*cKO mice at P1. **c** Co-immunolabeling of CC1 and Olig2 in the corpus callosum (CC), white matter (SC-WM), and gray matter (SC-WM) of the spinal cord of Ctrl and *Med23*cKO mice at P14. Scale bars: 100 μm. **d** Quantification of CC1^+^ OLs per area in the corpus callosum, white matter, and gray matter of the spinal cord of Ctrl and *Med23*cKO mice at P14. **e** Western blot analysis of myelin protein and GFAP levels in the cortex (CTX), hippocampus (HP), pons (PON), and cerebellum (CB) of Ctrl and *Med23*cKO mice at P14. β-Actin was used as the internal control. **f** Representative images showing cell morphological differences between Ctrl and *Med23*^*–/–*^ cells at the indicated differentiation stages as revealed by static staining. Scale bars: 50 μm. **g** Immunostaining for NG2 and CNP in Ctrl and *Med23*^*–/–*^ OPCs cultured in differentiation medium for 1 day and 3 days. White arrows indicate the CNP^+^NG2^–^ cells. Scale bars, 50 μm. **h** Quantification of CNP^+^NG2^–^ OLs as a percentage of total DAPI^+^ cells in the Ctrl and *Med23*^*–/–*^ OPC population that were cultured in differentiation medium in vitro for 1 day and 3 days. **i** Immunostaining for MBP, CC1, and Olig2 in Ctrl and *Med23*^*–/–*^ OPCs cultured in differentiation medium for 3 days. Scale bars: 50 μm. **j**, **k** The proportions of MBP^+^ (**j**) or CC1^+^ (**k**) OL differentiated from Ctrl and *Med23*^*–/–*^ OPCs cultured in differentiation medium for 3 days. The data are presented as the mean ± SEM; *n* = 3 animals/genotype or *n* = 3 independent experiments. Two-tailed unpaired Student’s *t*-test, *P < 0.05, **P < 0.01, and ***P < 0.001.

We wondered whether the hypomyelination phenotype in the *Med23*cKO mice is acquired because of OPC or OL generation failure. We first analyzed OPC formation by in situ hybridization at P1 (Fig. 4a). Comparable *Pdgfra*^+^ OPCs were observed in the control and *Med23*cKO spinal cords (Fig. 4b), suggesting that OPC specification proceeds normal in the absence of Med23. Moreover, the percentage of proliferative OPCs (as indicated by the percentage of proliferative EdU^+^Olig2^+^ OPCs) in the corpus callosum was similar between the *Med23*cKO and control mice (Supplementary Fig. S3a, b). These results indicated that deletion of Med23 did not alter OPC formation or proliferation.

We next observed a marked reduction in CC1^+^ mature OLs in the corpus callosum, spinal white matter and spinal gray matter of the *Med23*cKO mice compared with control at P14 (Fig. 4c, d). Similarly, the expression of MBP, a mature OL marker, was significantly lower in cortical regions, especially the superficial cortex, in the *Med23*cKO mice than that in the control mice (Supplementary Fig. S3c). Western blot analysis also revealed that myelin protein expression was greatly reduced in the CNS regions of the *Med23*cKO mice than in the control mice, which was consistent with the deficient myelin formation phenotype. However, no detectable GFAP expression difference between the *Med23*cKO and control mice was observed (Fig. 4e; Supplementary Fig. S3c), indicating that astrocytes differentiated normally. Together, these findings suggested that loss of Med23 in the OL lineage did not affect OPC formation and expansion but resulted in mature OL deficiency, which may have accounted for the hypomyelination pathology observed in the *Med23*cKO animals.

### Med23 deletion prevents OPC differentiation into fully mature OLs in vitro

To further investigate the role of Med23 in regulating the differentiation and/or maturation of OLs, we performed in vitro studies to monitor the growth and differentiation of primary OPCs that were dissociated from the brains of from the *Med23*cKO (i.e., *Med23*^*–/–*^ OPCs) or control mice (i.e., *Med23*^*+/+*^ OPCs). At OPC stage, there was no significant morphology difference between them, although the *Med23*^*–/–*^ OPCs exhibited a slightly reduced proliferation rate compared to the *Med23*^*+/+*^ OPCs, as indicated by the level of NG2^+^ and EdU^+^ cells (Supplementary Fig. S3d, e). These results, combined with our in vivo observations, suggested that Med23 deletion did not change the OPC identity.

After a period of OPC expansion, primary OPC cultures were differentiated by T3 treatment for 3 days. The static staining showed that most of the *Med23*^*+/+*^ OPCs extended numerous cellular processes to form a star-like morphology at the initial stage of process extension (T3 treatment for 1 day), whereas the great majority of *Med23*^*–/–*^ OPCs exhibited bipolar or tripolar OPC shapes (Fig. 4f). After 3d of T3 exposure, *Med23*^*–/–*^ OPCs exhibited significant deficits in their capacity to differentiate into typical arborized morphology. Consistently, after 1 day of T3 exposure, about 17% of the control OPCs had developed into CNP^+^NG2^–^ immature OLs; however, less than 1% of the *Med23*^*–/–*^ OPCs were immunopositive for CNP at this time point (Fig. 4g, h). Notably, after 3 days of T3 exposure, the control OPCs differentiated into fully arborized OLs, as indicated by CC1^+^ or MBP^+^ mature markers; however, the *Med23*^*–/–*^ OPCs exhibited significant deficits in the expression of these markers (Fig. 4i–k), even failed to express appreciable levels of CNP (Fig. 4g, h), suggesting that the *Med23*-null OPCs were intrinsically unable to enter the mature stage and appeared to be stalled at the initial stage of terminal differentiation. Collectively, these observations correlated closely with our findings in vivo, indicating that Med23 is required for full term OL maturation and that Med23 plays a primary role in the transition of OPCs into differentiated OLs, not OPC generation.

### Med23 controls the key gene programs for OL lineage progression

**Fig. 5:**
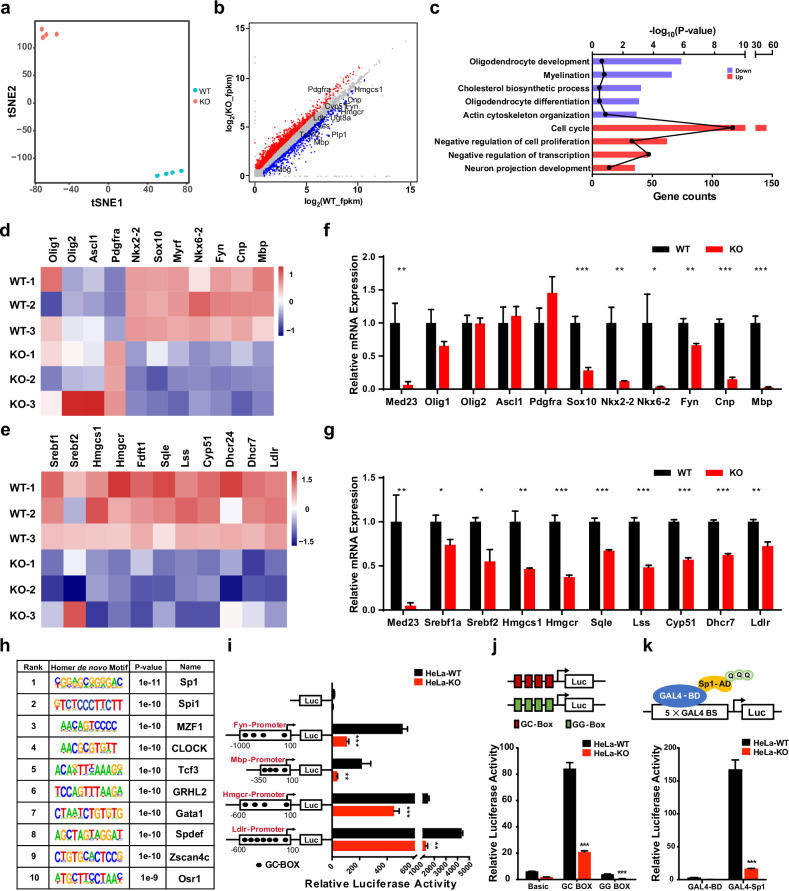
Med23 controls a regulatory network critical for OPC differentiation. **a** *t*-SNE analysis of RNA-seq data of *Med23*^*+/+*^ iOLs and *Med23*^*–/–*^ iOLs. **b** RNA-Seq transcriptome profiling changes between Ctrl and *Med23*^*–/–*^ iOLs. Representative transcripts of genes with downregulated expression (blue) or upregulated expression (red) are shown. **c** Gene ontology enrichment analysis showing the significantly altered genes between Ctrl and *Med23*^*–/–*^ iOLs. **d**, **e** Heatmaps showing the expression differences in OL differentiation-associated genes (**d**) and cholesterol metabolism-related genes (**e**) between Ctrl and *Med23*^*–/–*^ iOLs. **f**, **g** qRT-PCR analysis of OL differentiation-associated genes and cholesterol metabolic genes between Ctrl and *Med23*^*–/–*^ iOLs. Gene expression was normalized to that of 18S rRNA. **h** Enriched motifs in the promoter regions of significantly downregulated genes in *Med23*^*–/–*^ iOLs. **i** WT and *Med23*-KO HeLa cells were transfected with luciferase reporters driven by the *Fyn*, *Mbp*, *Hmgcr*, or *Ldlr* promoter together with expression vectors carrying Sp1. Values are presented as firefly luciferase activity normalized to co-transfected GFP fluorescence activity. **j** Sp1-targeted GC-box and GG-box reporter activity in WT and *Med23*-KO HeLa cells. **k** Sp1 activation domain fused to Gal4-DNA binding domain was tested for ability to activate transcription in WT and *Med23*-KO HeLa cells. The data are presented as the mean ± SEM; *n* = 3 independent experiments. Two-tailed unpaired Student’s *t*-test, *P < 0.05, **P < 0.01, and ***P < 0.001.

To characterize the role played by Med23 in gene expression during OL progression, we performed RNA sequencing (RNA-seq) analysis for the induced iOLs (T3 treatment for 1 day) derived from either *Med23*^*+/+*^ OPCs or *Med23*^*–/–*^ OPCs. First, as shown by *t*-SNE analysis, the *Med23*^*+/+*^ iOLs and *Med23*^*–/–*^ iOLs were clearly divided into two clusters distinguished by transcriptomic difference (Fig. 5a), implying that Med23 ablation caused substantial changes to intrinsic cellular programs during OPC differentiation. Global gene profiling showed that Med23 deletion resulted in a significant reduction of many OL development-related genes (Fig. 5b). Gene ontology analysis showed that downregulated genes were involved in biological processes related to myelination, cholesterol biosynthesis, and OL differentiation (Fig. 5c). Among these genes, a cohort of myelin genes and critical differentiation regulators, including *Fyn*, *Sox10*, *Nkx2-2*, *Nkx6-2*, *Cnp* and *Mbp*, were downregulated in the *Med23*^*–/–*^ iOLs. However, the expression of OPC specification factors *Olig1*, *Olig2*, and *Ascl1* was not significantly altered (Fig. 5d). These gene expression alterations were consistent with OL differentiation defects found in *Med23*^*–/–*^ iOLs. Interestingly, *Med23*^*–/–*^ iOLs also exhibited markedly reduced expression of a cluster of genes that encode enzymes involved in de novo cholesterol biosynthesis, including *Hmgcs1*, *Hmgcr*, *Fdps*, *Sqle*, *Lss*, *Cyp51*, *Dhcr7*, and *Ldlr*, a plasma cholesterol transport protein gene (Fig. 5e). These alterations were verified by qRT-PCR analysis with control and *Med23*^*–/–*^ iOLs (Fig. 5f, g). Importantly, the decreased expression gene sets related to OL differentiation and cholesterol metabolism in *Med23*^*–/–*^ iOLs were consistent with that in the optic nerves of the *Med23*cKO mice (Supplementary Fig. S4a). Consistently, transcriptional dysregulation of these genes was also observed in cells derived from Med23^Q649R^ mouse presenting with hypomyelination (Supplementary Fig. S4b). Overall, these in vitro data concurred with the in vivo data, indicating that Med23 plays a key role in orchestrating two classes of genes related to myelination and cholesterol metabolism that are both necessary for OL maturation.

### Mediator Med23 is a cofactor in the modulation of Sp1 activity

To investigate the transcription factors (TFs) that cooperate with Mediator Med23 in orchestrating multiple gene programs of OL differentiation, we applied the motif-discovery algorithm to identify TF-binding motifs enriched at the promoters of downregulated genes in *Med23*^*–/–*^ iOLs. The motif analysis showed considerable enrichment of binding motifs for several transcriptional regulators, and the Sp1-binding motif was the most overrepresented (Fig. 5h), suggesting that Sp1 is possibly involved in the regulation program.

Sp1 is a zinc finger transcription factor characterized by its affinity for GC-rich promoter sequences (GC-boxes)^25^. Several previous studies have implicated the involvement of Sp1 in activating the expression of a few myelination genes, such as *Fyn* and *Mbp*, as well as certain genes required for cholesterol homeostasis, including *Hmgcr* and *Ldlr*^26–29^. The four gene (*Fyn*, *Mbp*, *Hmgcr*, and *Ldlr*) promoters that harbor several Sp1-binding sites (indicated by ellipses in Fig. 5i) were all strongly stimulated by Sp1 overexpression; however, MED23 deficiency significantly impaired Sp1-driven reporter activity, suggesting the possibility that Mediator Med23 is required for Sp1 control of the expression of the aforementioned genes (Fig. 5i).

Pioneering work has revealed that Sp1 is a sequence-specific TF that recognizes GGGGCGGGG (GC-box), and a central G-substitution (GG-box) largely reduces Sp1 binding^30^. To verify the specific effect of Med23 regulation on Sp1-driven activation, we performed luciferase reporter assays with either the GC-box-Luc or GG-box-Luc reporter in control (HeLa-WT) and *MED23*-KO HeLa cells (HeLa-KO). Consistent with previous findings, Sp1 activated GC-box-Luc, but induced GG-box-Luc activity at a 22-fold lower level (Fig. 5j), suggesting that this reporter system can be used to monitor Sp1 transcriptional efficiency. We noticed that Med23 deficiency significantly impaired the Sp1-driven activation of GC-box-Luc and GG-box-Luc, although the latter showed very little response to Sp1 activation (Fig. 5j). Finally, to verify the direct effect of MED23 on Sp1 activity, Gal4-Sp1 fusion construct and Gal4-directed luciferase reporter were co-transfected into control and HeLa-KO cells. We found that MED23 depletion dramatically impaired the transactivation activity of Gal4-Sp1 (Fig. 5k). Together, these observations indicated that Mediator MED23 is a transcriptional co-activator that potentiates Sp1 transcriptional activity.

### Sp1 deficiency prevents OL differentiation

**Fig. 6:**
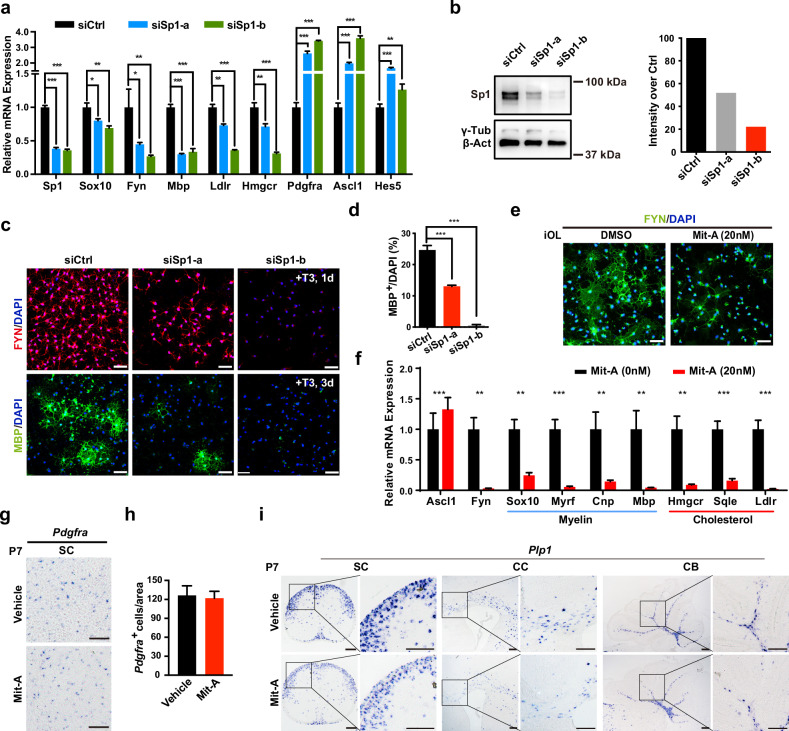
Inhibition of Sp1 expression reduces mature OL formation. **a** Relative expression of OPC markers (*Pdgfra*, *Ascl1*, and *Hes5*), OL differentiation-associated genes (*Sox10*, *Fyn*, and *Mbp*), and cholesterol metabolic genes (*Ldlr* and *Hmgcr*) as measured by qRT-PCR in siCtrl- and si*Sp1*-transfected OPCs after T3 treatment for 1 day. Gene expression was normalized to that of 18S rRNA. **b** Western blot analysis for Sp1 in siCtrl- and si*Sp1*-transfected OPCs, and α-Tubulin/β-actin were the internal controls. **c** Immunostaining for Fyn after 1 day of differentiation and MBP after 3 days of differentiation in siCtrl- and si*Sp1*-transfected OLs. Scale bars: 50 μm. **d** Quantifying the percentage of MBP^+^ cells relative to DAPI^+^ cells in siCtrl- and si*Sp1*-transfected OLs after 3 days of differentiation. **e** Representative images of immunostaining showing that Mit-A (20 nM) treatment for 24 h led to a dramatic reduction in Fyn expression in iOLs. Scale bars: 50 μm. **f** qRT-PCR analysis of the myelination-promoting genes in iOLs treated with DMSO or Mit-A. Gene expression was normalized to that of 18S rRNA. **g**, **h** In situ hybridization for *Pdgfra* (**g**) and quantification of *Pdgfra*^+^ OPCs (**h**) in spinal cords of mice that received DMSO or Mit-A treatment at P7. Scale bars: 100 μm. **i** In situ hybridization for *Plp1* in different brain regions of mice that received DMSO or Mit-A treatment at P7. Scale bars: 100 μm. The data are presented as the mean ± SEM; *n* = 3 independent experiments. Two-tailed unpaired Student’s *t*-test, *P < 0.05, **P < 0.01, and ***P < 0.001.

Although Sp1 has been found to be involved in activating some specific myelin genes^31,32^, it is not clear to what extent the Sp1 functions in OL development and myelination. To determine the Sp1 function, we first knocked down endogenous Sp1 expression in cultured mouse OPCs using short interfering RNAs (siRNAs) against *Sp1* (si*Sp1*). Scrambled siRNA served as the control (siCtrl), and the knockdown efficiency was confirmed by qRT-PCR and western blot (Fig. 6a, b). We found that si*Sp1* OPCs displayed no obvious deficiencies of the expression of OPC markers when cultured in the presence of PDGF (data not shown). Interestingly, after T3 differentiation medium incubation for 1 day, the siSp1 OPCs showed reduced expression levels of myelination-promoting genes, including *Fyn*, *Mbp, Ldlr*, and *Hmgcr*, compared to that of the OPCs transfected with the control siRNA. By contrast, the expression of several OPC-specific genes, such as *Pdgfra*, *Ascl1*, and *Hes5*, was somehow upregulated in Sp1-knockdown iOLs (Fig. 6a). Most si*Sp1*-treated cells failed to differentiate into MBP^+^ mature OLs even after T3 induction for 3 days; however, a number of siCtrl cells expressed high MBP levels (Fig. 6c). Remarkably, inhibition of Sp1 expression reduced the percentage of the MBP^+^ mature OLs, and the effect was dose-dependent (compared the effects between siCtrl, *siSp1-a*, and si*Sp1-b*, as shown in Fig. 6c, d), indicating that Sp1 was critical for activating myelination-promoting genes.

Mithramycin A (Mit-A), a clinically approved chemotherapeutic agent which competitively targets the chromatin-binding sites of Sp1, is usually used to interfere with Sp1 binding to its target sites^33^. We further analyzed the effect of Sp1 inhibition on OPC differentiation in vitro and observed that Mit-A significantly inhibited OPC differentiation, as indicated by the Mit-A-treated cells displaying reduced *Fyn* expression (Fig. 6e). Moreover, Mit-A treatment dramatically reduced the expression of cholesterol metabolic genes and differentiation-related genes (Fig. 6f), suggesting that inhibition of Sp1 function has disrupted the gene expression programs required for OL differentiation. We next asked whether Sp1 inhibition would disturb myelination in mice. Since Mit-A is known to be able to cross the blood-brain barrier^34^, we treated mice with 4 daily intraperitoneally administered injections of Mit-A from P3 to P6 to induce Sp1 inhibition and measured OL development in the white matter at P7. In situ, hybridization analysis showed that the expression levels of *Mbp* and *Plp1* were significantly reduced in the cerebral cortex, cerebellum, and spinal cord after Mit-A treatment (Fig. 6i), whereas the abundance of Pdgfra^+^ OPCs was comparable (Fig. 6g, h). These observations supported that Sp1 specifically regulates the OL differentiation process. Taken together, the data revealed a strong similarity with OL-development defects observed in the absence of either Med23 or Sp1 and the impact of MED23 on Sp1-driven activation suggested that they may act collaboratively at the same molecular nexus of regulatory networks to contribute to OL differentiation and myelination.

### Med23 modulates Sp1 cooperation with P300 in target gene expression

**Fig. 7:**
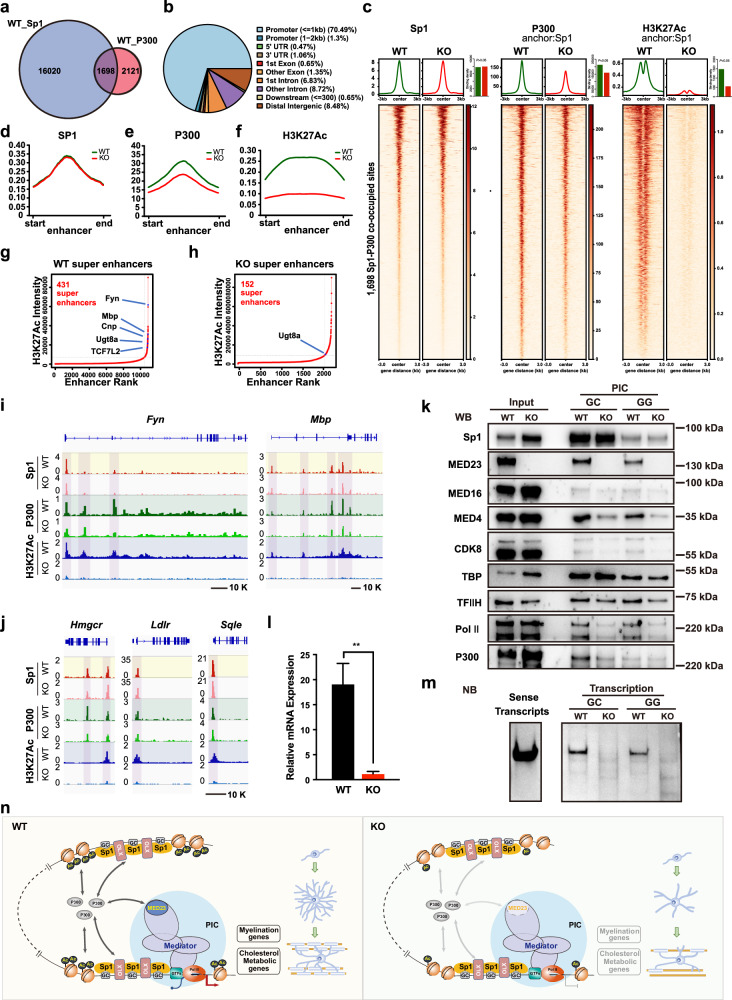
Med23 deletion leads to the loss of the Sp1-dependent P300-binding signal. **a** Venn diagram showing the genome-wide overlap between Sp1- and p300-bound gene sites in WT iOLs. **b** Pie chart presenting the genomic distribution of the co-occupied regions of Sp1 and P300 in WT iOLs. **c** The Sp1-binding density at the sites co-occupied by Sp1 and P300 (left) and the enrichment of P300 (middle) and H3K27Ac (right) centered around Sp1-binding sites in *Med23*^+/+^ iOLs and *Med23*^–/–^ iOLs. **d–f** Averaged ChIP-seq signals of Sp1 (**d**), P300 (**e**), and H3K27Ac (**f**) centered at active enhancer regions in *Med23*^*+/+*^ iOLs (green) and *Med23*^*–/–*^ iOLs (red). **g**, **h** “Hockey-stick” plots showing putative enhancers and SEs determined by H3K27Ac ChIP-seq in *Med23*^*+/+*^ iOLs (**g**) and *Med23*^*–/–*^ iOLs (**h**). The horizontal line represents the demarcation between typical enhancers (below) and SEs (above). **i**, **j** Interactive Genomics Viewer (IGV) browser views showing ChIP-seq for Sp1, P300, H3K27Ac at the representative loci. ChIP-seq was performed in *Med23*^+/+^ iOLs and *Med23*^*–/–*^ iOLs. The ChIP-seq signal was normalized to CPM (Counts Per Million mapped reads). **k** Western blot analysis of total Sp1 and other indicated factors bound to the DNA template in an immobilization assay with WT and *Med23*-KO HeLa cells. **l** In vitro transcribed RNA products from the GC template during the transcription step were analyzed by reverse transcription followed by qPCR quantification. **m** Northern blot of total RNA from transcription step of immobilized template assay. Probes are specific for the transcripts of DNA template. **n** A schematic model showing that controls OL lineage progression by facilitating Sp1-dependent P300 recruitment to enhance H3K27Ac deposition in lineage regulatory genes for promoting OPC differentiation. OLX is indicated as a potential lineage-specific factor that has not been identified in our study.

Previous studies indicated that Sp1 cooperates with histone acetyltransferases (HAT) P300 to control transcriptional activity in many cell lines^35–38^. However, whether Sp1 works with P300 during CNS myelinogenesis and the exact mechanism of their cooperative effects remain elusive. We first analyzed whether Sp1 and P300 co-regulate myelination gene expression and found that a combination of Sp1 and P300 overexpression promoted myelination genes expression, to a much greater extent than Sp1 overexpression alone (Supplementary Fig. S5a), suggesting that Sp1 and P300 may synergistically activate myelination-promoting genes. We next investigated whether Med23 affects the cooperation between Sp1 and P300 through performing a genomic occupancy analysis of Sp1, P300, and H3K27Ac in control and *Med23*^*–/–*^ iOLs. We observed that a substantial proportion of P300 peaks (1698 of 3819 total peaks) overlapped with those of Sp1 binding in WT iOLs (Fig. 7a). Genomic distribution analysis of the 1698 co-occupied peaks showed that they were enriched in the promoter regions (71.7%) and other noncoding regions (Fig. 7b). The heatmaps showed no difference of Sp1-binding levels at the 1698 colocalized sites between control and *Med23*^*–/–*^ iOLs, suggesting that Med23 seems not to interfere with Sp1-binding (Fig. 7c, left panel); however, Med23 deficiency resulted in a reduction (∼24%) in P300 occupancy (Fig. 7c, middle panel) and a substantial decrease (∼62%) in H3K27Ac enrichment (an active chromatin modification by P300) surrounding the 1698 Sp1 binding sites (Fig. 7c, right panel). By contrast, the P300-binding signals at Sp1-free regions (2121 of 3819 total peaks) showed no difference between control and *Med23*^*–/–*^ iOLs (Supplementary Fig. S5b), underscoring that Med23 is selectively important for Sp1-dependent P300 occupancy and H3K27Ac modification.

It is well established that the H3K27Ac mark, deposited by p300, is an important histone mark that decorates both active enhancers and promoters that generally correlated with gene activation^39–41^. By defining enhancers through the H3K27Ac signal density at noncoding regions co-occupied by Sp1 and P300, we observed that Med23 deletion led to a marked decrease in P300 and H3K27Ac enrichment at the putative enhancers (Fig. 7e, f), suggesting diminished enhancer activity, whereas Sp1 binding was unaffected by the presence or absence of Med23 (Fig. 7d). Super-enhancers (SEs) are defined as large clusters of enhancers that are associated with hyperactive genes that define cell identity^42^. We next asked whether Med23 impacts the establishment of H3K27Ac-marked SEs correlating with OL identity. H3K27Ac chromatin immunoprecipitation sequencing (ChIP-seq) data were then interrogated via the ROSE algorithm^43^ for identification of SEs in iOLs. Analysis of enhancer landscapes revealed more than 10,000 enhancers in WT iOLs, among which 431 were classified as SEs, and some of them were associated with critical myelin genes, such as *Fyn*, *Cnp*, *Mbp*, *Ugt8a*, and *Tcf7l2* (Fig. 7g); however, in the *Med23*^*–/–*^ iOLs, the total number of enhancers was greatly reduced (to ~2000), and the SEs associated with the aforementioned myelinogenic genes were diminished (Fig. 7h). Analysis of specific loci also revealed a loss of P300 and H3K27Ac enrichment at the regulatory elements of myelination-promoting genes in *Med23*^*–/–*^ iOLs, including *Fyn*, *Mbp*, *Hmgcr*, *Ldlr*, and *Sqle* (Fig. 7i, j). Together, these results suggested that Mediator Med23 was critical for preserving H3K27Ac levels of Sp1/P300-associated typical enhancers and SEs, which contribute to cell identity change during OL lineage progress.

Lastly, to biochemically dissect how Mediator Med23 impacts on the Sp1-P300 collaboration in transcription, we utilized the immobilized template assay to analyze the preinitiation complex (PIC) assembly and subsequent transcription. In this assay, the biotinylated template containing the 4 tandem classical Sp1-binding motifs (GC-box or GG-box) was incubated with nuclear extracts (NEs) derived from either WT or *Med23*-KO HeLa cells. The preinitiation complex (PIC) was formed by incubating bead-immobilized DNA templates with nuclear proteins, while the subsequent addition of nucleoside triphosphate (NTP) mix could trigger the transcription (depicted in Supplementary Fig. S5c). We first observed that GG-template dramatically reduced the Sp1 binding compared with GC-template, and the amount of Mediator (indicated by MED4, MED16, CDK8 subunits), RNA Pol II, and P300 that immobilized on the GG-template were consistently compromised (Fig. 7k, compare lanes 3 and 4 with 5 and 6). This system allowed us to monitor the factors that were recruited in a Sp1-directed manner. We next assessed the effect of MED23 depletion on recruitment of P300 and other components and observed that MED23 depletion did not affect the binding ability of Sp1at GC template, which was consistent with the *Sp1* genomic occupancy analysis performed with the control and *Med23*^*–/–*^ iOLs (Fig. 7c). TBP were also detected at a comparative level that is not affected by the presence or absence of MED23. However, attenuated binding of P300 was observed in *Med23*-KO HeLa NEs (Fig. 7k), suggesting that MED23 is required for the P300 recruitment. In addition, it seems that the MED23 deficiency also led to reduced Mediator significantly. We reasoned that the Mediator is recruited by Sp1 in a MED23-dependent manner, although the physical interaction between MED23 and Sp1 was not detected by co-immunoprecipitation (co-IP) assay (data not shown). Noticeably, the RNA Pol II enrichment at the PIC stage was largely reduced in the absence of MED23 (Fig. 7k). We therefore asked whether the Sp1-dependent transcription was affected by the MED23. The qRT-PCR analysis showed that the transcripts synthesized from the immobilized template in the *MED23*-KO HeLa NEs is far less than that in WT HeLa NEs (Fig. 7l). Consistently, Northern blotting showed that the generated transcripts decreased sharply in the absence of MED23, underscoring the critical role for Mediator Med23 in Sp1-dependent PIC formation and active transcription (Fig. 7m). Collectively, our data indicated that Mediator Med23 was a key component in orchestrating the collaboration between Sp1, Mediator, P300, and GTFs/Pol II in driving the OL lineage-specific gene transcription (Fig. 7n).

## Discussion

In our study, the functional importance of the Mediator Med23 in myelin development and related neurological diseases was revealed by utilizing the cellular differentiation system and lineage-specific Med23 inactivation mouse model. Combining stage-specific RNA-seq with genomic occupancy analyses, we delineated the molecular mechanisms involving Mediator Med23, transcription factor Sp1, and HAT P300 in controlling gene programs that are essential for OL maturation, myelination, and remyelination after injury.

Cholesterol is the major component of myelin membranes, and the cholesterol incorporation process is critical for myelin production and maintenance^44–46^. Therefore, de novo cholesterol synthesis and uptake by OLs must be highly synchronized with lineage progression. However, how the cholesterol metabolism coordinates with OL differentiation is not well understood. Our results revealed that the expression of many classical OL-differentiation genes was significantly reduced in the absence of Med23 (Fig. 5d), and mechanistically, Med23 regulates OL maturation by directly controlling these myelin gene expressions. In the meantime, Med23 also played an important role in maintaining adequate cholesterol for CNS myelinogenesis, as Med23 deletion led to dramatic alterations in almost all the genes responsible for cholesterol biosynthesis and uptake (Fig. 5e). Previous studies have shown that insufficient cholesterol could block OL maturation and myelinogenesis^45,47^. We therefore postulated that the requirement of Med23 for OL myelination could be partly mediated through its role in cholesterol metabolic during OL maturation. Significantly, in this study we identified Mediator Med23 as a previously unknown key regulator that integrates gene programs for both OL-differentiation and cholesterol metabolism during OL maturation.

In recent years, P300/CBP activity has been directly implicated in enhancer activation, and P300/CBP-catalyzed H3K27Ac is widely used as a marker of activated enhancers that mediate the transcription of cell-type-specific genes^43^. Human genetic studies have shown that loss-of-function mutations in the EP300 gene, which encodes HAT P300, cause Rubinstein-Taybi syndrome, which is manifested as hypoplasia of the corpus callosum, congenital hypomyelination, and other systemic abnormalities^48^, suggested that P300 may serve as a vital regulator in OL development. A recent study reported that HDAC3 interacts with P300 to regulate OL-astrocyte fate switching^49^. However, how P300 regulates the transcriptional program in OL lineage development has not been fully explained. In the present study, by performing a gain-of-function study, we verified that P300 was required for the activation of Sp1-targeted myelination-promoting genes. Moreover, the iOL stage-specific CUT&Tag analysis of Sp1 and P300 verified their cooperation in activating genes related to OL differentiation and cholesterol metabolic processes. Notably, our integrative analysis of the Sp1-P300-H3K27Ac enrichment profile revealed the requirement of P300 in the SEs establishment, which correlated with the expression of OL cell fate determination genes, including *Fyn*, *Cnp*, *Tcf7l2, and Mbp* (Fig. 7g). Thus, we demonstrated a novel mechanism through which P300 participates in regulating OL development, thus providing insights into the pathogenesis of Rubinstein-Taybi syndrome.

Importantly, we identified Mediator Med23 as a coregulator in modulating the Sp1-dependent P300 recruitment and activity of target genes. This discovery aligns with a series of findings showing that Mediator may act as a multifunctional integrator that orchestrates various TFs and cofactors at different stages of transcription^7,50,51^. In particular, the Mediator complex regulates the PIC assembly process by directly binding to P300^52^, and P300 can be recruited to enhancers by Mediator^53^. All these observations are complementary to our findings showing that Med23 deletion led to Mediator defects in recruiting P300 to Sp1 target genes. We further demonstrated the specific functional interplay between Mediator Med23, Sp1, and P300, in which Med23 influenced only P300 occupancy and activities in a Sp1-dependent manner; whereas Med23 deletion did not alter the P300-binding signals at Sp1-free regions (Supplementary Fig. S5b).

Clinical genetic studies have revealed that most individuals with *MED23* mutations suffer from ID and other developmental disorders, with a subset diagnosed with hypomyelination features, such as pontine hypoplasia and thinning corpus callosum. Inspired by these studies, we established the Med23^R649Q^ mutation in a rodent model and characterized the histological, cellular, and molecular consequences of this mutation. Notably, in addition to the hypomyelination phenotypes, Med23^Q649R^ mice showed worsened working memory in behavior tests (Fig. 1n, o), manifesting cognitive impairment. These findings support the notion that the common pathophysiology of many patients with cognitive decline may partially originate from abnormal CNS myelination. However, the neurodevelopmental disorders caused by MED23 mutations in patients, including microcephaly and global developmental delay, appear to be more severe than those in Med23^Q649R^ mice. We reasoned that Med23 mutations in other cell types, such as neurons or astrocytes, may contribute to the human phenotype. Functional analysis of Med23 in the development of neurons and other glial cells awaits future studies.

In this study, we also observed that the Med23 deficiency compromises the OL regeneration and remyelination. The OL-specific *Med23*-KO mice were utilized to monitor the difference in myelin repair, as we considered that the Med23-deficiency does not change much of the OPC development but impacts the OL differentiation; and the local injection of LPC in the white matter induces rapid myelin breakdown in either control or *Med23*cKO mice, which provides the equal basis for comparing the remyelination process between the WT and *Med23*cKO mice. Nevertheless, it would be ideal to use the inducible *Med23*-KO mice model for the remyelination experiment in the future.

In summary, we identified Mediator Med23 as a previously unknown regulator in myelin development and revealed novel molecular mechanisms for OL cell fate determination, providing potential therapeutic targets for demyelinating diseases.

## Materials and methods

### Animal studies

All animals were maintained under a 12-h light/12-h dark cycle in specific pathogen-free conditions. All animal experiments were performed in accordance with the guidelines of the Institutional Animal Care and Use Committee of the Shanghai Institute of Biochemistry and Cell Biology.

*Med23*-floxed (*Med23*^*fl/fl*^) mice were generated as described previously^14^. *Med23*-floxed mice were crossed to transgenic *Olig1-cre* mice^15^ to construct OL lineage conditional KO mice (*Med23*^*fl/fl*^*;Olig1-cre*, i.e., *Med23*cKO).

Med23^Q649R^ mice breeding and genotyping. CRISPR-mediated homology-directed repair was used to generate C57BL/6 mouse model with point mutation (Q649R), which is parallel to the human pathogenic mutation (GRCh38.p13; chr6:131603042T>C; c.1937A>G; p.Q646R). A Cas9 gRNA (Seq: 5’-ACCACAGATGGAGCTGGTTCTGG-3’) was designed to target the 17th exon of the mouse *Med23* gene. And the single strand oligonucleotides carrying homology to the targeted region and the G mutation was used as donor (Seq: 5′-CGAGTTCAGCTCCTGAGCCATCTCCACACACTGGCTGCAGTCGCACAGACCAACCAGAACCGGCTCCATCTGTGGTGAGTGAGCAGCCTGCAGGCTGCCTCCCCTAGGGTTTAGTCCACCCAT-3′). Founders carrying a mutant allele (Med23^+/Q649R^) were generated, but the homogeneous Med23^Q649R/Q649R^ mutant mice is embryonic lethal. We crossed the heterozygous mice (Med23^+/Q649R^) with *Med23*^*fl/fl*^*;Olig1-cre* mice to generate OL lineage-specific *Med23* mutant mice (*Med23*^*fl/ Q649R*^*;Olig1-cre*,), and their littermates (*Med23*^*fl/+*^*;Olig1-cre*) were used as experimental control. Subsequent genotyping at each generation was conducted utilizing allele specific PCR with mutation flanking primers: forward primer 5′- GCTAACTGGTCCCTGGAATT-3′ and reverse primer 5′- TTCCAAGCATGGGTGGACTA-3′. PCR products were purified using the FastPure Gel DNA Extraction Mini Kit (Vazyme) according to the manufacturer’s instructions. Purified PCR products were then sent for Sanger sequencing with the responsive forward primer 5′-ATCCTGCACACACTGCTG-3′.

### Cell lines

HeLa cell lines were described previously^54^. All cell lines were cultured in DMEM supplemented with 10% fetal bovine serum (FBS), 100 U/mL penicillin, and 100 μg/mL streptomycin at 37 °C with 5% CO2.

### Primary OPC isolation and culture

Primary OPCs were purified by immunopanning as the protocol^55^. Briefly, brains were obtained from P7 and diced, followed by digestion in papain at 37 °C for 90 min. Dissociated cell mixture were incubated on BSL1-coated plates twice to deplete endothelial cells and microglia. Then the remaining cells were immunopanned on CD140a antibody-coated petri dishes for OPCs selection. After rinsing non-adherent cells away, immunopanned OPCs were removed from the final panning dishes with trypsin and transferred to poly-D-lysine-coated tissue culture dishes or glass coverslips in 12-well tissue culture plates. Isolated OPCs were grown in Sato growth medium supplemented with mitogens 10 ng/mL PDGF-AA and 1 ng/mL NT-3. For inducing differentiation, the medium switched to OL differentiation medium (Sato medium supplemented with 15 nM T3 and 10 ng/mL ciliary neurotrophic factor) for 1 and 3 days to become iOL and mOL as the initiation and maturing phases of OLs, respectively, as previously described^56^. OPCs were transfected with knockdown siRNAs or expression plasmids using Amaxa Nucleofector (Lonza) according to the manufacturer’s protocol.

### Electroporation of OPCs

Cultured OPCs were passaged by washing the plates once with EBSS (equilibrated in 10% CO2 incubator) and then treated the plate for 8 min at 37 °C with trypsin-EBSS solution. Cells were collected with 30% FBS/DPBS, and centrifuged at 2500 rpm for 10 min. Cells were resuspended and counted in cold DPBS and centrifuged for another 15 min to remove traces of trypsin. The cell pellet was resuspended to 5 × 10^7^ cells/mL in OPC Amaxa Nucleofector solution (Lonza). Either expression plasmids (~ 4 μg) or siRNAs (10 μL × 20 μM) were added to 100 μL (5 × 10^6^ cells) cell suspension and electroporated with the Amaxa Nucleofector apparatus on program O-017. Cells were then plated out at 20,000 cells/PDL-coated coverslip in 12 well plates or at 3 × 10^5^ cells/ PDL-coated well of 6-well-plate in proliferative medium and shifted to the OL differentiation medium one day after transfection and maintained for 72 h.

### EM

EM was performed as described previously^57^. Briefly, mice were euthanized and perfusion-fixed with 2.5% (v/v) glutaraldehyde and 2.0% (w/v) paraformaldehyde (PFA) in 0.1 M cacodylate buffer (pH 7.4). Spinal cord and optic nerves were removed into the same fixative, kept overnight at 4 °C, and transferred into 0.1 M cacodylate buffer. After immersion in a 1% (w/v) osmium tetroxide solution for 2 h at 4 °C, the specimens were dehydrated through a graded alcohol series and embedded in Epon 812. Ultrathin sections were stained with lead citrate for electron microscopy imaging. The g-ratio is defined as the ratio of the inner axonal diameter to the entire fiber diameter. Blind counts of myelinated axons were performed on images of randomly selected areas of the optic nerves and spinal cords. At least 100 axons of 0.3 μm or greater diameter were counted from 5 images per sample.

### Tissue processing and immunofluorescence

Mice were euthanized followed by transcardial perfusion with ice-cold PBS and 4% PFA. Brains and spinal cord were dissected, fixed in 4% PFA overnight, dehydrated in 30% sucrose at 4 °C, embedded in OCT, and cryo-sectioned at 20 μm. For immunofluorescence, cryo-sections or cell culture on PDL-coated coverslip were fixed with 4% PFA in PBS for 10 min and then permeabilized, blocked in blocking solution (0.01% Triton X-100 and 3% BSA in PBS) for 1 h at room temperature. Primary antibodies (Supplementary Table S1) were then applied overnight at 4 °C. After washing three times with PBS for 5 min, sections or cells were incubated with the appropriate fluorophore-conjugated secondary antibodies for 2 h at room temperature. Fluorescent images were captured by a digital camera using Zeiss-LSM880 confocal microscope. The images were quantified in a double-blinded manner.

### In situ hybridization

In situ hybridization was performed as described previously^58^. Briefly, digoxigenin (DIG)-labeled RNA probes were transcribed in vitro from cloned cDNAs for mouse *Pdgfra*, *Mbp*, and *Plp1*. For single-probe in situ hybridization, the DIG signal was visualized with alkaline phosphatase (AP)-conjugated anti-DIG Fab2 fragments and a mixture of nitroblue tetrazolium (NBT) and 5-bromo-4-chloro-3-indolyl phosphate (BCIP).

### LPC-induced demyelinating injury

LPC-induced demyelination was performed in the ventrolateral spinal white matter of ~8-week-old mice as described previously^56^. Mice were euthanized, the spinal vertebrae were exposed at the level of T9–T12, and meningeal tissue in the intervertebral space was cleared. 0.5 μL of 1% LPC (Sigma L4129) via a syringe attached to a glass micropipette was injected into the ventrolateral white matter via a stereotactic apparatus. Mice were left to recover in a warm chamber before being returned to their cages. Spinal cord tissues carrying the lesions were collected at day 7 or day14 after injection. The experiments were conducted in a genotype-blinded manner.

### Static staining

Cell static staining was performed according to manufacturer’s instructions. Briefly, remove cell medium and incubate the cells in 4% PFA solution for 15 min at room temperature. Wash fixed cells 2–3 times with PBS and add 0.1% Triton X-100 solution to permeabilize cells for 15 min at room temperature. Wash the cells 2–3 times with PBS. 100 μL of HCS CellMask staining solution (10 mg/mL) was added to each well for incubation for 30 min at room temperature. Each well was washed with PBS 2–3 times. Images were taken under confocal microscopy Zeiss-LSM880 with appropriate filters.

### Luciferase reporter assay

The dual-luciferase reporter assay was performed as described^59^. Promoter regions for analysis of Sp1 binding were chosen using the Eukaryotic Promoter Database (https://epd.epfl.ch//index.php). Promoter regions were amplified by PCR and inserted into pGL3-luc (Promega) as upstream of the luciferase gene. The HeLa cell line was used for luciferase reporter gene assays. Cells were transiently transfected with a luciferase reporter plasmid and pRL-TK (Promega) by Lipofectamine 2000. All wells were supplemented with control empty expression vector plasmids to keep the total amount of DNA constant. The cells were harvested and subjected to dual-luciferase reporter assays after 24–36 h of transfection according to the manufacturer’s protocol.

The Sp1-responsive luciferase reporter plasmid constructed from pGL3-Basic Vector with 4 tandem repeats of a typical GC-box or GG-box inserted into multiple cloning sites upstream of firefly luciferase reporter gene. This construct monitors both increases and decreases in the transcription activity of Sp1. The Gal4 luciferase reporter vector driven by four *Gal4* DNA-binding sites has been described previously^14^. The Gal4-Sp1 constructs were created by fusing the sequence encoding transactivation domain A and B (amino acids 148–497) of mouse Sp1 (https://www.uniprot.org/uniprot/O89090) to the sequence encoding *Gal4* DNA binding domain. The in-frame fusion of the cDNA fragment with the *Gal4* DNA-binding domain was confirmed by sequencing.

The constitutively expressing GFP plasmid acts as an internal control for normalizing transfection efficiencies and monitoring cell viability. The expression of the GFP from the positive control construct can be monitored by fluorescence microscopy using an excitation filter of 470 ± 20 nm and an emission filter of 515 nm.

### In vitro basal transcription assays with immobilized template and Northern blotting

The method for the immobilized template assay is based on the previous study^60^ with some modifications. In brief, two DNA templates labeled with biotin at the upstream end were generated by PCR from pGL3-GC box or pGL3-GG box plasmid used in luciferase reporter assays. The templates were 200 bp and contained 4 repeats GC- box followed by Luciferase CDS. Then, 6 μg of each biotinylated template was bound to 300 μg Dynabeads M-280 Streptavidin (Dynal) according to the manufacturer’s instructions with the aid of a DynalMagTM-2 (Dynal). Immobilized templates were resuspended in 100 μL of block buffer (20 mM HEPES, pH 7.9, 0.1 M KCl, 10% glycerol, 6 mM MgCl2, 2.5 mM DTT, 50 mg/mL BSA). After a 15-min incubation at room temperature, 100 μL of WT or MED23 KO HeLa nuclear extract in Buffer D (20 mM HEPES, pH 7.9, 100 mM KCl, 8 mM MgCl2, 10% glycerol, 0.5 mM DTT, 0.5 mM PMSF), was incubated for 30 min at 30 °C to allow PIC formation. Immobilized templates were washed 3 times with buffer D plus 0.1% Triton X-100. Proteins bound to immobilized templates were eluted with SDS gel loading buffer and subjected to 8% SDS-PAGE. The primary antibodies used in the western blot are listed in the Supplementary Table S1. For in vitro transcription, NTPs mix (25 mM each) was added to stimulate transcription after PIC formation and the reaction was incubated at 30 °C for 1 h. The supernatant which contains RNA products was collected for northern blotting.

Northern Blotting analysis was performed according to the previous study^61^ with minor modification. In brief, Digoxigenin (Dig)-labeled antisense riboprobes were made using DIG RNA labeling KIT (Roche). Total RNA samples were collected from in vitro transcription assays with the GC-box or GG-box DNA templates and loaded into an 8% TBE urea gel (Bio-Rad) and transferred to a nitrocellulose membrane (Beyotime). The dried membrane was UV-cross-linked and then exposed to a prehybridization solution for 1 h. The membrane was then hybridized with specific Dig-labeled RNA probes. NB probes were in vitro transcribed from the following DNA template with *T7* promoter (5′- CATTCCGGTACTGTTGGTAAAGCCACCATGGAAGACGCCAAAAACATAAAGAAAGGCCCGGCGCCATTCTATCCGCTGGAAGATGGAACCGCTGGAGAGCAACTGCATAAGGCTATGAAGAGATACGCCCTGGTTCCTGGAACAATTGCTTTTACAGATGCACATATCGAGGTGGACATCACTTACGCT -3′).

### Y-maze test

The Y-maze test was conducted as described before^62^ with minimal changes. The Y-maze apparatus consisted of three light-colored, opaque arms orientated at 120 ° angles from each other (40 × 5 cm with 10 cm high walls). Spontaneous alternation behaviors were assessed. Bring mice into the testing room at least 30 min prior to the test to for acclimatization. The test mouse was allowed to explore the Y-maze for 8 min freely, and arms choices (all four paws entering one arm) were recorded by a camera. Maze arms were cleaned with 70% ethanol solution between trials to remove any olfactory cues. Overlapping triplets of 3 arm visits were counted as one complete spontaneous alternation. The score was calculated as the following formula: (number of spontaneous alternation)/(total number of arm visits – 2) × 100%.

### NORT

The test was modified from previous protocols^19^. Briefly, the whole test was a 3 days trial, consisting of habituation, training, and test stage. Bring mice into the testing room at least 30 min for acclimatization on two consecutive days prior to the testing day. In the training stage, mouse is allowed to explore two identical objects placed in the experimental chamber (40 cm × 40 cm × 40 cm) for 5 min. Then the mouse is kept individually for 1 h in a new cage until the test starts. During retention, the chamber and objects were cleaned with 70% ethanol to remove fecal and urine. In the test stage, substitute one of the familiar objects (F) with a novel object (N). The mouse was allowed to explore in the experimental chamber for 10 min. The time in exploration of different objects (F or N) in the test stage was calculated. Record the amount of time that the mouse spends in investigating each object Observer 4.1 Software (Noldus). The preference for novel object was calculated as the following formula: Recognition Ratio = exploration time of novel object/(time in exploration of novel object + time in exploration of familiar object) × 100.

#### RNA extraction, qRT-PCR and RNA-seq analysis

Tissue or cells were lysed in TRIzol followed by phenol-chloroform extraction. cDNA libraries were prepared using HiScript III RT SuperMix for qPCR (Vazyme). RT-qPCR were performed using SYBR qPCR Master Mix (Vazyme) on QuantStudio 7 Flex Real Time PCR System. The level of the endogenous mRNA was normalized to the level of 18S mRNA using 2^–△△^^CT^ method. qRT-PCR primers are available upon request. RNA-seq raw reads were obtained from illumina X-10 sequencing analyzer. All sequencing reads were QC checked with FastQC. Adapters and poor-quality bases were trimmed using Cutadapt. Clean reads were aligned to mouse reference genome mm10 using Tophat2 with default parameters. Genes and transcripts quantification and differential expression analysis were performed by cuffdiff with parameters -b -u. Differentially expressed genes were identified with fold changes of more than 2 of expression level. We used findMotifs.pl of HOMER software to analyze the promoters of differentially expressed genes and looked for motifs that are enriched in these gene promoters. Functional enrichment analysis was performed using online website DAVID (https://david.ncifcrf.gov/) and barplots were plotted in GraphPad Prism.

### ChIP and ChIP-seq

ChIP-seq was performed as previously described^54^. Briefly, cells were fixed and sonicated into ~300–500 bp using a COVARIS S220 sonicator. IP was performed overnight at 4 °C with 2–4 μg of antibody. Antibody-bound chromatin was isolated on protein G magnetic beads and DNA was finally purified with phenol-chloroform. ChIP-seq libraries were generated with VAHTS Universal DNA Library Prep Kit for Illumina V2 (Vazyme Biotech, ND607) and sequenced on the Illumina Hi-Seq Xten.

### CUT&Tag library construction and data processing

CUT&Tag was performed with Hyperactive In-Situ ChIP Library Prep Kit for Illumina (pG-Tn5) (Vazyme Biotech, TD901) following the manufacturer’s instructions. Briefly, Concanavalin-A beads was washed and resuspended in wash buffer before collecting cells. 100,000 cells for each library were suspended in 100 μL wash buffer. Pre-treated ConA beads was added to each sample and incubated for 10 min at room temperature. Beads with cells were isolated and resuspended with Antibody buffer, and then the primary antibody was added and incubated for 2 h at room temperature on a rotator. Then beads were isolated with a magnetic stand and suspended in Dig-wash Buffer-diluted secondary antibody solution. After 60-min incubation, the beads were washed three times with Dig-wash buffer and incubated with Hyperactive pG-Tn5 in Dig-300 Buffer for 60 min. Afterward, beads were washed twice with Dig-300 buffer and resuspended in tagmentation buffer for incubation. To stop the tagmentation reaction, 10 μL of 0.5 M EDTA, 3 μL of 10% SDS, and 2.5 uL of 20 mg/mL Proteinase K were added to samples and incubated at 50 °C for 1 h. finally, DNA was extracted with phenol-chloroform and ethanol precipitation. All DNA was subjected to PCR to amplify the libraries. All libraries were sequenced by Illumina Hi-Seq Xten.

### Quantification and statistical analysis

For qPCR and luciferase assay, an average value was calculated from at least three independent replicates. For statistical analysis of positive immunostaining cells and in situ hybridization signals, an average percentage was calculated from at least 3 different fields from at least three independent experiments. GraphPad Prism software was used to determine statistically significant differences. The data were presented as the means ± SEM in the graphs. Student’s *t*-test was used to compare the effects of different two groups. Differences were considered statistically significant at *P < 0.05, **P < 0.01, ***P < 0.001.

## Supplementary information

Supplementary Information

## Data Availability

The datasets generated during this study have been deposited in the Gene Expression Omnibus public database under accession numbers GSE226121, GSE226122, and GSE226123.
