# Supplementary material for: Mediator MED23 controls oligodendrogenesis and myelination by modulating Sp1/P300-directed gene programs
Source: Cell Discov. 2024 Oct 15;10:102. doi: 10.1038/s41421-024-00730-8 (PMC11473658; doi:10.1038/s41421-024-00730-8)
Supplement: Supplementary file 1 — Supplementary Information [file 41421_2024_730_MOESM1_ESM.pdf]

Supplementary Information

Mediator MED23 controls oligodendrogenesis and myelination by modulating Sp1/P300-directed gene programs

Shuai Zhang, Xue Feng, Chong-Hui Li, Yuan-Ming Zheng, Meng-Ya Wang,

Jun-Jie Li, Yun-Peng Dai, Naihe Jing, Jia-Wei Zhou, Gang Wang*

*Correspondence: gwang_fd@fudan.edu.cn

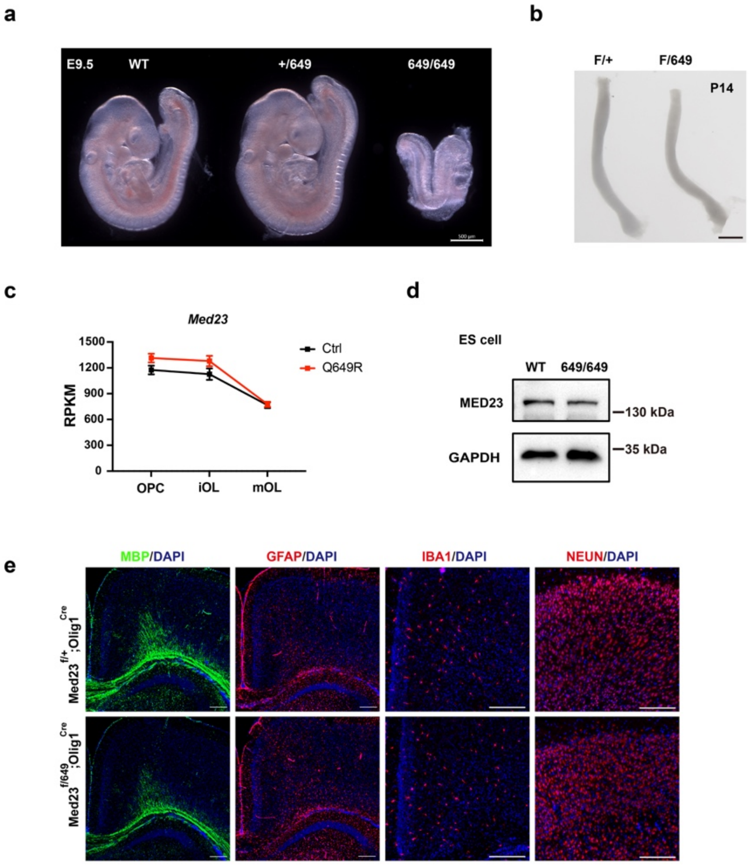

Supplemental Fig. S1 Med23^{Q649R} mutation did not affect OPC formation, related to Fig. 1. a Whole mount E9.5 embryos at the same magnification. Left: wildtype; Middle: Med23$^{+/Q649R}$ mutant; Right: Med23$^{Q649R/Q649R}$ mutant. Scale bar, 500 µm. **b** Appearance of optic nerves from Med23$^{f/+}$ and Med23$^{f/Q649R}$ mutant mice at P14. Scale bar, 1 mm. **c** RNA-seq analysis of Med23 transcripts in primary mouse OPCs, iOLs, and mOLs. Data are presented as mean ± SEM, n = 3 at each time point. **d** Western blot analysis of ES cells from WT and 649/649 mutants with anti-MED23 (amino-terminal epitope) and GAPDH as the loading control. **e** Immunostaining for MBP and GFAP in the corpus callosum, Iba1 and NeuN in the cortex of control and Med23^{Q649R} mice at P14. Scale bars, 100 µm.

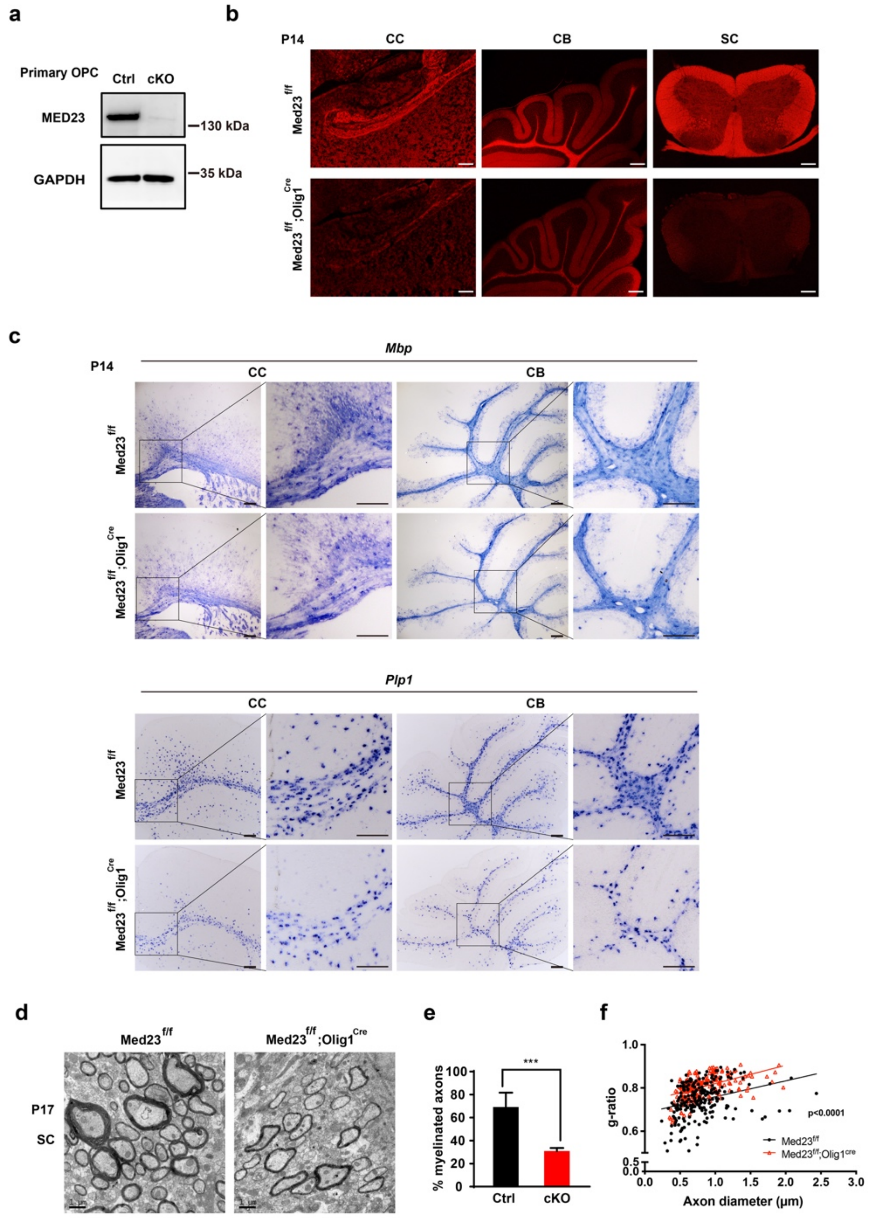

Supplemental Fig. S2 Med23 is critical for CNS myelination, related to Fig. 2. a Western blot analysis of primary OPCs isolated from Ctrl and Med23cKO brains with anti-MED23 (amino-terminal epitope) and GAPDH as the loading control. **b** Representative images showing *FluoroMyelin* staining of key white matter regions (corpus callosum, cerebellum and spinal cord) of Ctrl and Med23cKO mice at 2 weeks old. Scale bars, 100 μm. **c** *In situ* hybridization for *Mbp* and *Plp1* in the spinal cord from Ctrl and Med23cKO mice at P14. Scale bars, 100 μm. **d** Representative electron micrographs showing the spinal cord in Ctrl and Med23cKO mice at P17. Scale bar, 1 μm. **e** The myelinated axon percentage of Ctrl and Med23cKO optic nerves at P28 and P60. The data are presented as the means ± SEM; n =3 animals/genotype. Two-tailed unpaired Student's t test. ***p < 0.001. **f** Myelin g-ratio scatterplot of Ctrl and Med23cKO optic nerves at P28 and P60, n = 3 animals/genotype. (≥70 myelinating axons were counted for each mouse). Two-tailed unpaired Student's t test. p < 0.0001.

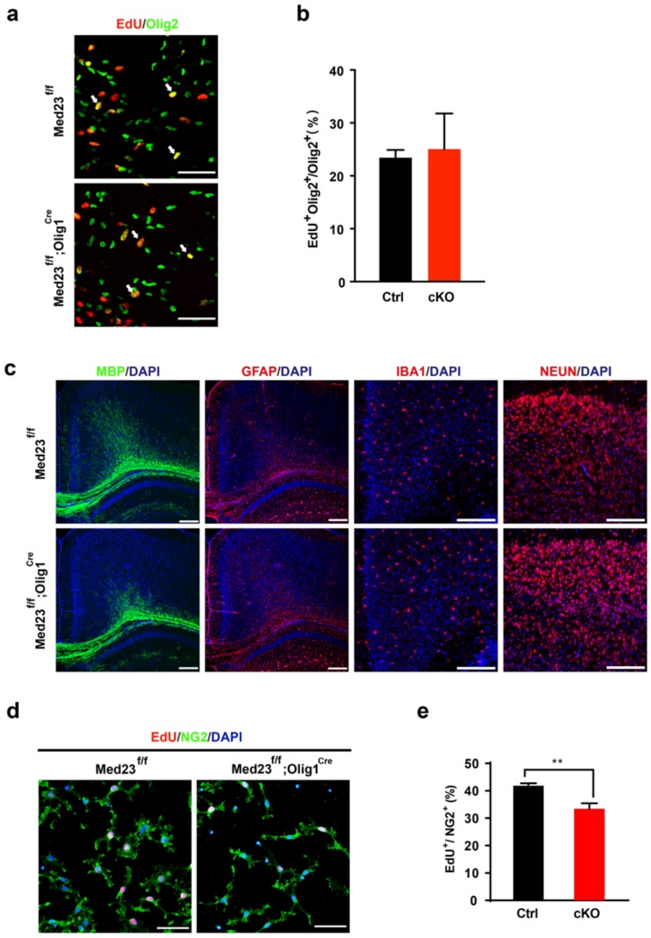

Supplemental Fig. S3 Med23 deletion leads to a reduction in mature OLs but not OPCs, related to Fig. 4. a Immunostaining for EdU and NG2 in the corpus callosum of P7 Ctrl and Med23cKO mice. Scale bars, 50 µm. **b** Quantification of EdU$^+$Olig2$^+$ OPCs as a percentage of Olig2$^+$ cells in the corpus callosum of P7 Ctrl and Med23cKO mice. The data are presented as the means ± SEM; n=3 independent experiments. P =0.7022, two-tailed unpaired Student's t test. **c** Immunostaining for MBP and GFAP in the corpus callosum, Iba1and NeuN in the cortex of Ctrl and Med23cKO mice at P14. Scale bars, 100 µm. **d** Immunostaining for EdU and NG2 in Ctrl and Med23$^{-/-}$ OPCs cultured with PDGF-AA in vitro. Scale bars, 50 µm. **e** Percentage of EdU$^+$ OPCs in Ctrl and Med23$^{-/-}$ OPCs cultured with PDGF-AA in vitro.

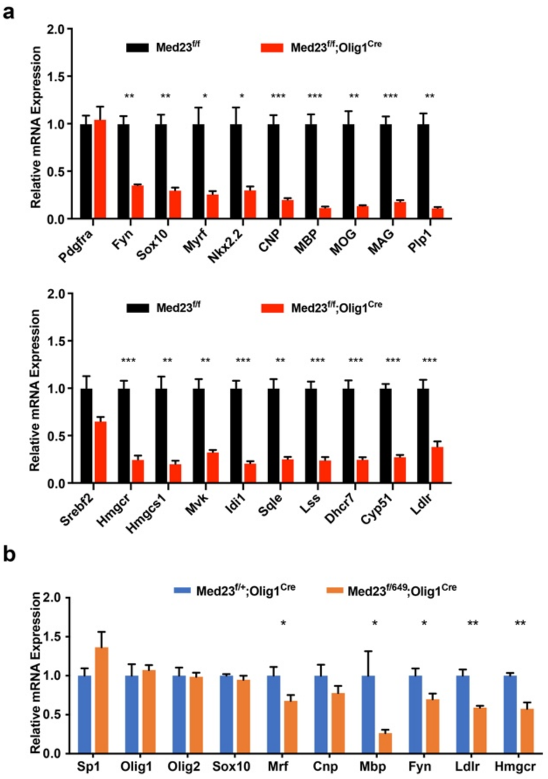

Supplemental Fig. S4 Med23 acts as a coregulator of Sp1, related to Fig. 5. a qRT-PCR analysis of the mRNA levels of OL differentiation-associated genes and cholesterol metabolism-related genes in the optic nerves of Ctrl and Med23cKO mice at P14. **b** qRT-PCR analysis of the mRNA levels of OL differentiation-associated genes and cholesterol metabolism-related genes in OPCs of control and Med23^{Q649R} OLs after 3 days of differentiation.

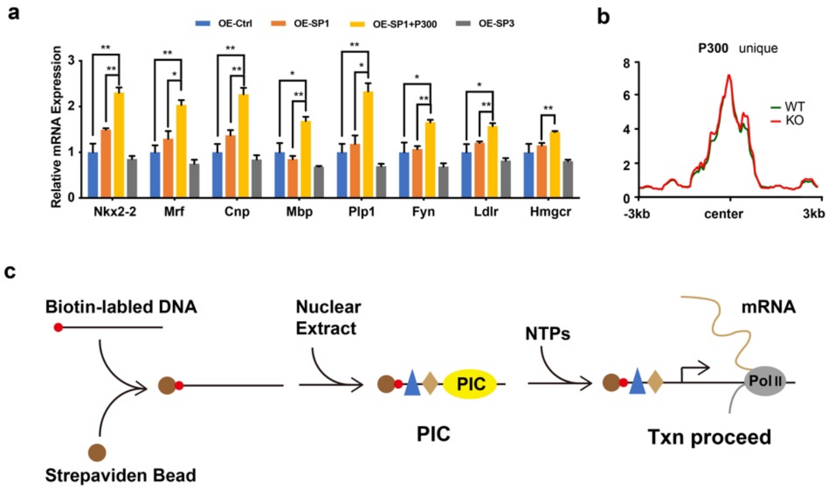

Supplemental Fig. S5 Sp1 cooperates with P300 to activate myelination-promoting genes, related to Fig. 7. a qRT-PCR analysis of OL differentiation genes and cholesterol metabolism genes in mouse OPCs transfected with Sp1, p300, or both Sp1 and p300 or Sp3 under differentiation conditions. **b** The signal density of P300 peaks plotted relative to Sp1-free regions in Med23$^{+/+}$iOLs (green) and Med23$^{-/-}$iOLs (red). **c** Schematic showing the procedure for immobilized assay.

Supplementary Tables

Supplementary Table S1 List of antibodies used in this study

Protein	Company	Catalog No.	Source
CD140a	BD Biosciences	558774	Mouse monoclonal antibody
MBP	Millipore	05-675	Mouse monoclonal antibody
CNP	Millipore	MAB326R	Mouse monoclonal antibody
CC1	Millipore	OP80	
GFAP	Millipore	MAB360	Mouse monoclonal antibody
NG2	Millipore	AB5320	Rabbit polyclonal antibody
OLIG2	Millipore	AB9610	Rabbit polyclonal antibody
MOG	Millipore	Mab5680	Mouse monoclonal antibody
Sp1	Millipore	07-645	Rabbit polyclonal antibody
Fyn	Abcam	Ab125016	Rabbit monoclonal antibody
MED23	Abcam	Ab200351	Rabbit monoclonal antibody
P300	SantaCruz	SC-585	Rabbit polyclonal antibody
H3K27ac	Abcam	ab4729	Rabbit polyclonal antibody
TFIIH	SantaCruz	SC-292	Rabbit polyclonal antibody
Sp1	Abcam	Ab227383	Rabbit polyclonal antibody
TBP	SantaCruz	SC-273	Rabbit polyclonal antibody
MED4	SantaCruz	SC-398179	Mouse monoclonal antibody
Pol II	SantaCruz	SC-56767	Mouse monoclonal antibody
GAPDH	Proteintech	60004-1	Mouse monoclonal antibody
γ-Tubulin	Proteintech	15176-1-AP	Rabbit polyclonal antibody
β-Actin	Proteintech	66009-1-Ig	Mouse monoclonal antibody
MED16	Abcam	Ab130996	Rabbit polyclonal antibody
CDK8	Abcam	Ab229192	Rabblt monoclonal antibody
